# S100A8/A9 Stimulates Keratinocyte Proliferation in the Development of Squamous Cell Carcinoma of the Skin via the Receptor for Advanced Glycation-End Products

**DOI:** 10.1371/journal.pone.0120971

**Published:** 2015-03-26

**Authors:** Guergana Iotzova-Weiss, Piotr J. Dziunycz, Sandra N. Freiberger, Severin Läuchli, Jürg Hafner, Thomas Vogl, Lars E. French, Günther F. L. Hofbauer

**Affiliations:** 1 Department of Dermatology, University Hospital Zurich, Zurich, Switzerland; 2 Institute of Immunology, University Clinic Münster, Münster, Germany; University of Miami, UNITED STATES

## Abstract

Squamous cell carcinoma (SCC) is the most common neoplasm in organ transplant recipients (OTR) on long-term immunosuppression and occurs 60- to 100-fold more frequently than in the general population. Here, we present the receptor for advanced glycation end products (RAGE) and S100A8/A9 as important factors driving normal and tumor keratinocyte proliferation. RAGE and S100A8/A9 were transcriptionally upregulated in SCC compared to normal epidermis, as well as in OTR compared to immunocompetent patients (IC) with SCC. The proliferation of normal and SCC keratinocytes was induced by exposure to exogenous S100A8/A9 which in turn was abolished by blocking of RAGE. The migratory activities of normal and SCC keratinocytes were also increased upon exposure to S100A8/A9. We demonstrated that exogenous S100A8/A9 induces phosphorylation of p38 and SAPK/JNK followed by activation of ERK1/2. We hypothesize that RAGE and S100A8/A9 contribute to the development of human SCC by modulating keratinocyte growth and migration. These processes do not seem to be impaired by profound drug-mediated immunosuppression in OTR.

## Introduction

Squamous cell carcinoma is a common skin neoplasm characterized by infiltrative, destructive growth and metastasis. It is the most common malignant neoplasm in organ transplant recipients on long-term immunosuppression and occurs 60- to 100-fold more frequently than in the general population [1]. The early recognition of SCC is important because the neoplasm may acquire the ability to metastasize. Actinic keratoses (AKs) are considered by some as precancerous lesions, while others consider them an incipient form of SCC [2]. Studies have demonstrated that approximately 8% of all AKs will progress to invasive SCC in the general population [3], and potentially more in OTR. Recognition and treatment of AK are important for the prevention of neoplasm progression. It is well known that AK is surrounded by a peritumoral inflammatory infiltrate before development of invasive SCC, also observed in OTR under immunosuppression [4–5]. Anti-tumour defence by the immune system seems to play an important role on one side. On the other side, chronic sustained inflammation seems to create a pro-tumorigenic environment [6]. Such smoldering inflammatory mechanisms in the skin may be at least in part mediated by RAGE and S100A8/A9 [7–10].

RAGE is a multi-ligand member of the immunoglobulin superfamily of cell surface molecules [11–13] and is implicated in inflammation and cancer [14–18]. RAGE ligation activates important signal transduction pathways involved in tumorigenesis and inflammatory responses such as the mitogen activated protein kinase (MAPK) family (p38, Erk1/2 and JNK) and Rho GTPases (cdc42 and rac) [19–22]. To the spectrum of RAGE ligands belongs the S100 family of proteins (calgranulins) including S100A12 [23], S100A9 [24–26] and S100A8/A9 heterodimer [19, 27, 28]. They activate cellular processes and cell migration, and have properties similar to proinflammatory cytokines [29–32]. S100A8 and S100A9 are secreted by neutrophils and activated monocytes [33–34] and induce activation of NF-κB [31, 35, 36]. They are associated with chronic inflammation [37–39] and cancer [10, 39–43].

Up to now the role of RAGE-S100A8/A9 signaling in keratinocytes in SCC formation has not been investigated. Here, we analyze the involvement of these proteins in the development of human SCC.

## Results

### RAGE, S100A8 and S100A9 are expressed in SCC of OTRs and IC

#### Epidermal mRNA expression of S100A8 and S100A9 differs between OTR and IC with invasive SCC

**Fig 1:**
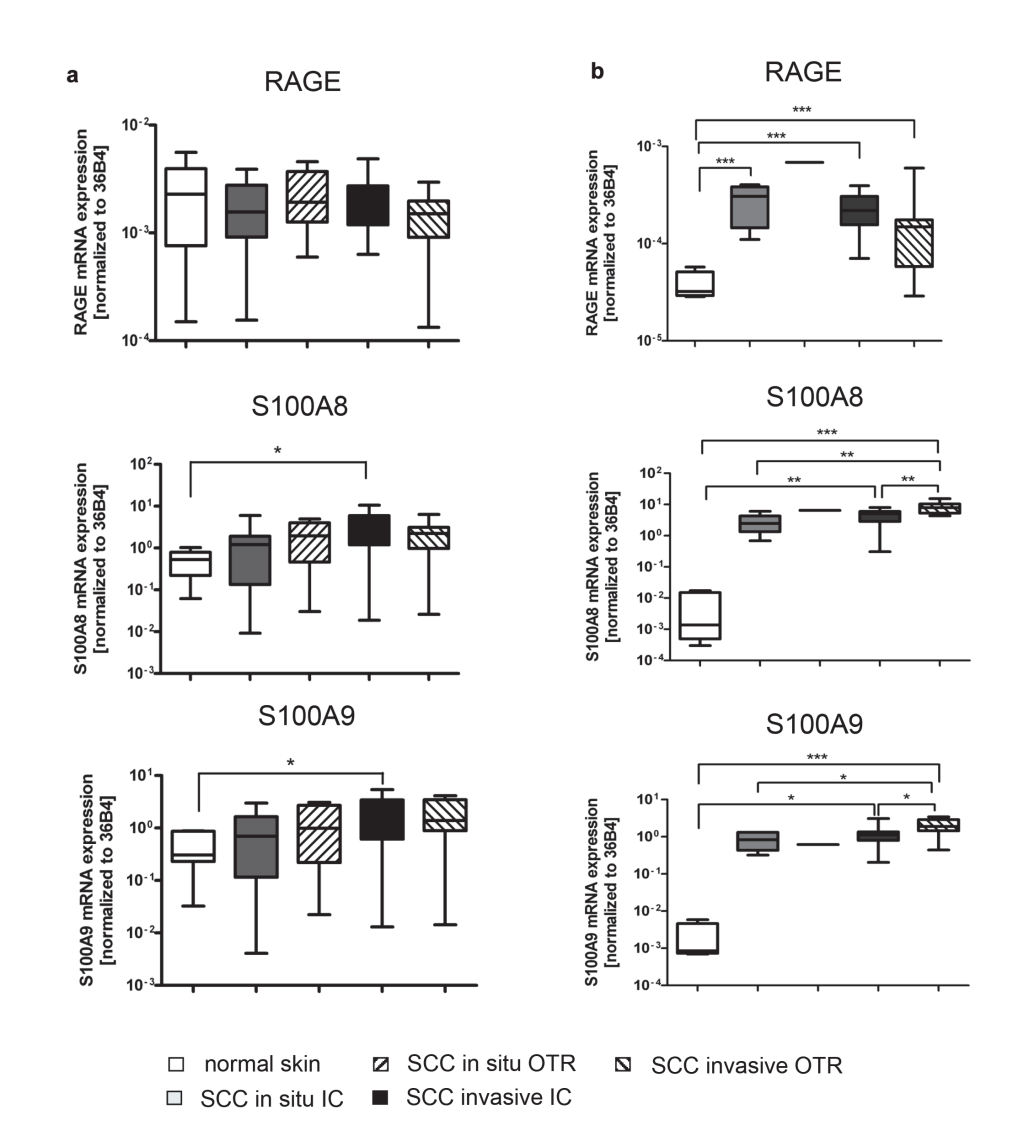
Differential expression of RAGE, S100A8 and S100A9 on transcriptional level in whole skin and epidermis. Expression of RAGE, S100A8 and S100A9 on transcriptional level. The expression was assessed by real-time PCR using specific primers for RAGE, S100A8 and S100A9. The expression level was normalized to 36B4 housekeeping gene. a: whole skin: No significant difference of RAGE expression was detected within or among the groups. S100A8 and S100A9 mRNAs are increased only in IC patients with invasive SCC versus normal skin (*p<0.05). b: epidermis: The expression of RAGE, S100A8 and S100A9 transcripts was significantly increased in OTR and IC patients with invasive or in situ SCC versus normal epidermis (***p<0.001). Significant difference in the expression of S100A8 and S100A9 was also detected between the IC and OTR groups with invasive SCC (*p<0.05; **p<0.01).

The mRNA expression level of RAGE in whole skin was similar in normal skin, IC SCC and OTR SCC. S100A8 and S100A9 mRNA expression was increased only in SCC of IC versus normal skin (Fig. 1A). In the epidermal fraction, however, the expression of RAGE, S100A8 and S100A9 was increased in invasive as well as in in-situ SCC of IC and OTR, all compared to normal epidermis (Fig. 1B).

#### Expression of RAGE and S100A8/A9 in SCC and normal skin on protein level

**Fig 2:**
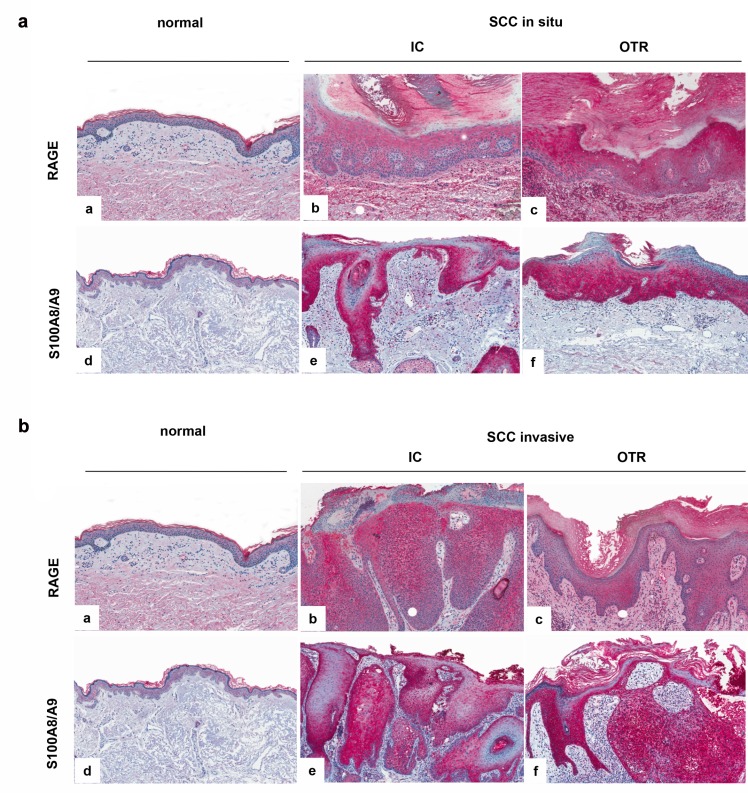
Differential expression of RAGE and S100A8/A9 on protein level in whole skin. The expression level was analysed by immunochistochemistry using specific anti human S100A8/A9 and anti human RAGE antibodies. The expression was tested in 5 patient samples per group (IC and OTR with in situ or invasive SCC). a: Expression of RAGE and S100A8/A9 in IC and OTR patients with in situ SCC in comparison to normal skin. b: Expression of RAGE and S100A8/A9 in IC and OTR patient with invasive SCC in comparison to normal skin.

The expression of RAGE and the S100A8/A9 complex on protein level was analyzed by immunohistochemistry. Although the analysis is semi-quantitative, both proteins were clearly upregulated in invasive or in situ SCC of IC and OTR in comparison to healthy skin (Fig. 2). We did not detect a differential protein expression of RAGE and S100A8/A9 between the OTR and IC groups.

### Endogenous S100A8/A9 is involved in cellular proliferation

**Fig 3:**
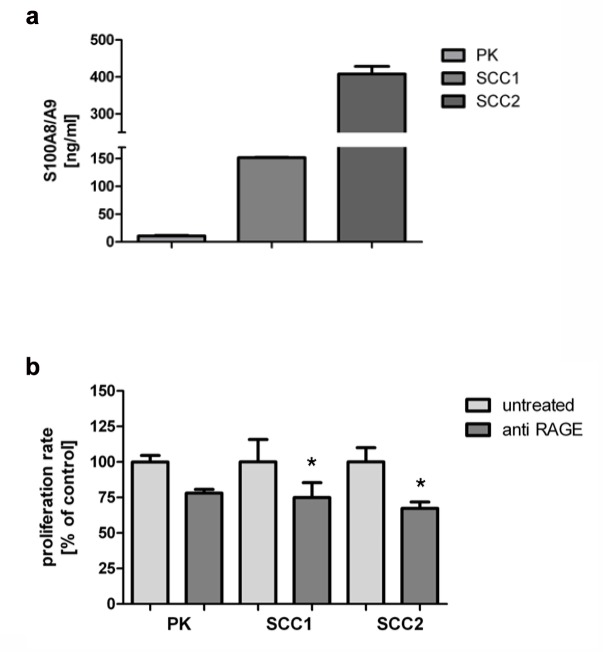
Endogenous S100A8/A9 is involved in cellular proliferation. a: Spontaneous secretion of S100A8/A9 from normal and SCC-derived keratinocytes. Normal and SCC-derived keratinocytes were grown in 96 well plates for 24 hours. Afterwards, supernatant was collected and preceded for the assessment of secreted S100A8/A9 using specific ELISA for S100A8/A9. b: Blockade of RAGE using anti RAGE blocking antibody reduces spontaneous proliferation of keratinocytes. Normal and SCC-derived keratinocytes were incubated with a blocking anti-RAGE antibody (8ug/100μl) for 24 hours. A decrease of the proliferation rate was detected (20–30%) based on the BrdU incorporation (t-test *p = 0.002). All the results are presented as percentage deviation from corresponding control and represent the mean +/- SD of duplicate values.

Using ELISA specific for S100A8/A9, we detected that normal keratinocytes secrete S100A8/A9. In comparison to SCC-derived keratinocytes, normal keratinocytes showed lower levels of spontaneous S100A8/A9 secretion (Fig. 3A). The direct blockade of RAGE using a specific neutralizing anti-RAGE antibody resulted in a reduction of cellular proliferation by 20–25% (Fig. 3B). This suggests that the endogenous production of S100A8/A9 and RAGE signaling contributes to keratinocyte proliferation.

### Keratinocytes proliferate in response to exogenous S100A8/A9

**Fig 4:**
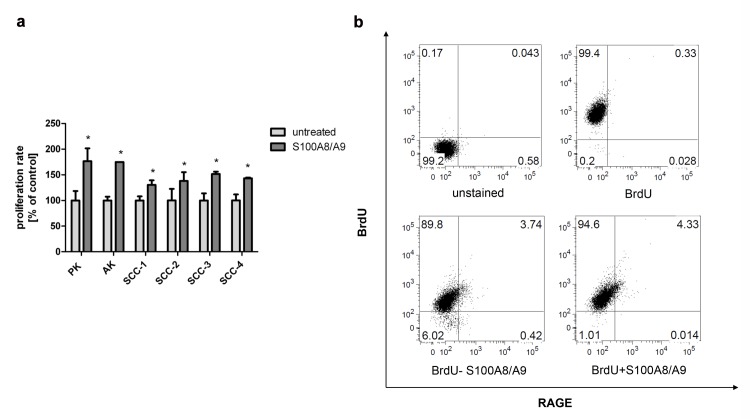
Exogenous S100A8/A9 induces keratinocyte proliferation. a: Assessment of the proliferation by BrdU assay. To assess the role of S100A8/A9 in normal primary, AK and SCC cultures, they were treated with purified S100A8/A9 for 24 hours and proliferation was assessed by BrdU incorporation. The induction of cellular proliferation was between 30–70% (2 way Anova, p<0.0001). b: Assessment of the proliferation by the incorporation of BrdU analysed by flow cytrometry. Normal keratinocytes were treated with S100A8/A9 (10ng/ml) and BrdU for 24 hours. Afterwards cells were fixed in 4% PFA, permeabilized with 0.5% Triton and stained for RAGE and BrdU using the antibodies mentioned above. The differential BrdU incorporation was compared to untreated cell.

Cells were seeded in 96 well plates and exposed for 24 hours to purified S100A8/A9 at concentrations between 0.01–1μg/ml. All normal, AK and SCC-derived primary keratinocytes responded with increased proliferation ranging from 30% to 70% after 24 hours exposure to S100A8/A9 as revealed by BrdU assay (Fig. 4A). Induced BrdU incorporation by S100A8/A9 was also detected by flow cytometry (Fig. 4B). The total RAGE expression was analyzed by FACS and western blotting (S1 Fig. A, B).

### Direct blockade and knockdown of RAGE reduce cellular proliferation

**Fig 5:**
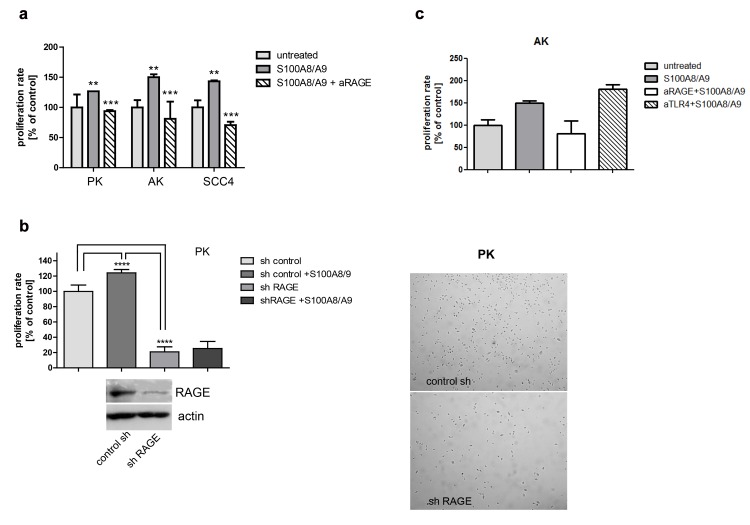
The receptor RAGE critically mediates the cellular response to S100A8/A9. a: Direct blockade by a specific RAGE blocking antibody reduces cellular proliferation. RAGE dependent proliferation was analyzed in normal primary, AK and SCC cells. Cells were incubated for 1 hour with a blocking anti-RAGE antibody (80μg/ml, as recommended by manufacturer) followed by S100A8/A9 stimulation for additional 24 hours. The differences in the proliferation after the blockade were assessed by BrdU incorporation (1 way Anova, Bonferroni`s Multiple test, **p<0.05, ***p<0.05). b: Knockdown of RAGE using shRNA reduces cellular proliferation. The knockdown studies were performed using specific lentiviral shRNA against RAGE. Primary keratinocytes were infected by shRAGE and sh control viral particles. Selection of positive clones was performed by puromycine selection. Cells were grown to optimal confluence and were stimulated with 10ng/ml S100A8/A9.Cells with RAGE knockdown showed a delay in proliferation in comparison to control as assessed by BrdU (70%) (1 way Anova, Bonferroni`s Multiple test p****<0.0001) and microscope images. Exogenous S100A8/A9 did not induce proliferation of shRAGE keratinocytes, but only in the control. c: Blocking TLR4 using specific blocking antibody (HTA125) does not impair cellular proliferation. AK cells were treated with a specific blocking TLR4 antibody (HTA125). AK cells were grown for 24 hours in the presence of HTA125 antibody (1μg/ml) and S100A8/A9 (10ng/ml). For detection of the cellular proliferation rate BrdU proliferation assay was performed.

Keratinocyte proliferation following S100A8/A9 exposure was abolished by blockade of RAGE using a functional blocking antibody (Fig. 5A). A reduction of cellular growth by 80% was detected also when shRNA specific for RAGE was used (Fig. 5B). Proliferation was not influenced by stimulation of shRAGE-transfected keratinocytes with recombinant S100A8/A9. Using the antibody HTA125 blocking TLR4, a potentially important receptor for S100A8/A9, proliferation was not impaired following exposure of keratinocytes to S100A8/A9 (Fig. 5C).

### S100A8/A9 induces migration of normal and SCC-derived keratinocytes

**Fig 6:**
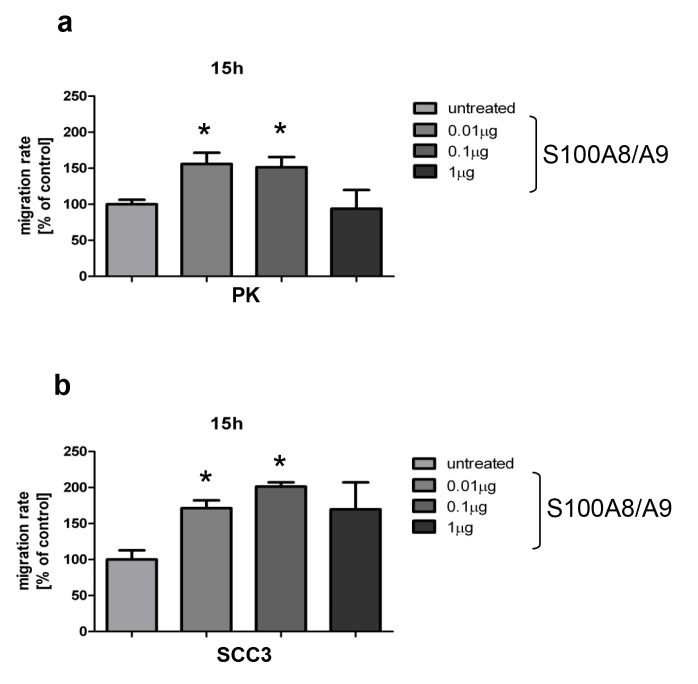
Exogenous S100A8/A9 induces migration of normal and SCC-derived primary keratinocytes. a, b: Cells were treated with different concentrations of purified S100A8/A9 (0.01–1 μg/ml) for 15h. The assessment of the migratory potential was analyzed by scratch assay where the number of migrated cells was analyzed. The migration of both normal and SCC-derived keratinocytes was increased significantly (between 60–100% depending on cell type) when 0.01 and 0.1μg/ml of S100A8/A9 were used (t-test, PK, *p = 0.003, *p = 0.004; SCC, *p = 0.0014,*p = 0.0015).

The effect of S100A8/A9 on the ability of normal and SCC-derived keratinocytes to migrate *in vitro* was investigated using scratch assay. An increase in the number of migrated cells was detected 15 h after scratching (between 60–100%) in both normal and SCC-derived keratinocytes in the presence of 0.01 and 0.1μg/ml S100A8/A9 (Fig. 6 A, B). At 24 hours after scratching, the difference was no longer observed.

### Exogenous S100A8/A9 induces RAGE surface expression and MAPK phosphorylation

**Fig 7:**
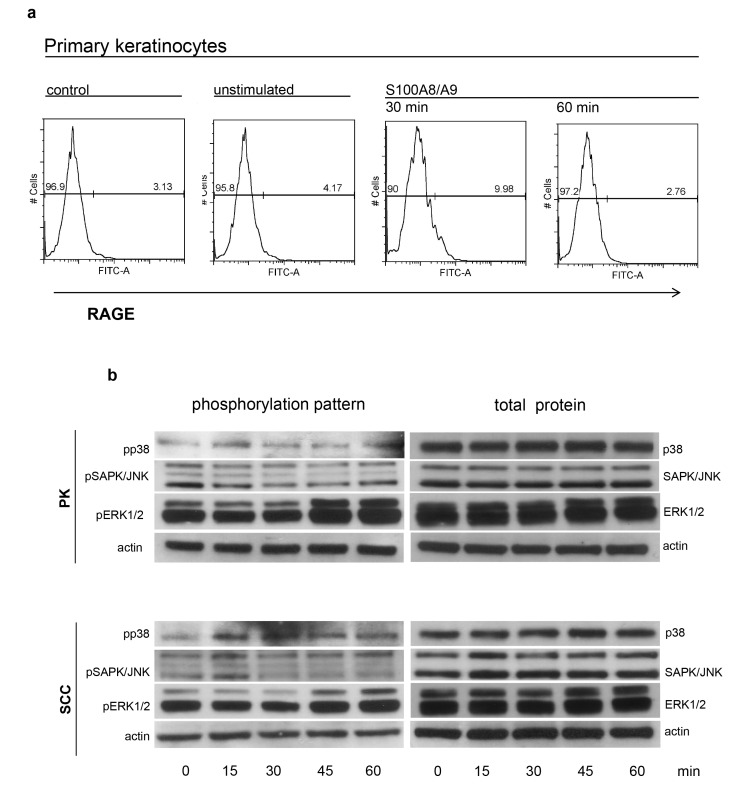
Exogenous S100A8/A9 induces RAGE surface expression and MAPK phosphorylation. a: Rapid and transient induction of RAGE surface expression after stimulation with S100A8/A9. Normal keratinocytes were induced with an exogenous S100A8/A9 (10ng/ml) in time intervals of 30 and 60 minutes. Afterwards cells were stained with the specific goat polyclonal anti RAGE antibody and a secondary FITC-conjugated antibody for analysis of both surface and total RAGE expression before and after S100A8/A9 treatment. Total expression of RAGE was analyzed prior to cell permeabilization with PFA and followed by blocking step with 1% BSA. As controls isotype control antibody and untreated cells were used. b: Exogenous S100A8/A9 induces phosphorylation of MAPKs. Normal and SCC-derived keratinocytes were seeded in 6cm dishes in 50% confluence. Cells were starved for 18 h and stimulated afterwards with S100A8/A9 (10ng/ml) for different time intervals (0–60min). Cell lysates were collected after each time interval and the alteration of the phosphorylation of ERK1/2, p38 and SAPK/JNK was analyzed by western blot using the indicated antibodies.

Exogenous S100A8/A9 induced rapid and transient increase in RAGE surface expression 30 min after S100A8/A9 stimulation and reduced it 1 hour after induction (Fig. 7A), which underlines the stimulation of S100A8/A9 through RAGE. To clarify the mechanism of S100A8/A9 we further investigated the phosphorylation of p38, ERK1/2 and JNK/SAPK after stimulation with S100A8/A9 (Fig. 7B). SCC-derived keratinocytes showed a prolonged activation of p38 phosphorylation in comparison to normal keratinocytes whereas the phosphorylation of p38 was slightly decreased 15 min after treatment. The phosphorylation of ERK1 decreased slightly 15–30 min after exposure to S100A8/A9 in both PK and SCC in comparison to unstimulated cells (Fig. 7B, S2 Fig.). An increase in the phosphorylation rate of ERK 1 was detected at later time intervals (45–60 min). SCC cells reacted with a phosphorylation of SAPK/JNK 15 minutes after exposure to S100A8/A9, whereas in normal keratinocytes phosphorylation of SAPK/JNK was not detected.

## Discussion

Normal whole skin and fully excised SCC express RAGE, S100A8 and S100A9 mRNA in similar amounts, with only minor increases for S100A8 and S100A9 in IC SCC, while other inflammatory factors such as NFkB, CCL3, CCL2, Cox2, TGFß and MMP9 do differ from normal skin to SCC (data not shown). This is probably due to the heterogeneous cell population present in whole skin. As reported, S100A8 and S100A9 are predominantly secreted by myeloid cells, but also by epithelial cells and keratinocytes during inflammation [27, 32, 33, 44–46]. In the epidermal compartment, however, both RAGE and S100A8 and S100A9 were strongly upregulated at mRNA and protein level already in intraepithelial lesions, suggesting an early role in SCC development. No additional difference was observed when comparing normal epidermis and SCC from IC and OTR patients, which suggests a role for RAGE and S100A8/A9 not impaired by commonly used immunosuppressive drugs. Previous studies in our group demonstrated that immunosuppressive agents such as Prednisolone and Cyclosporine are even able to induce S100A8/A9 expression in keratinocytes [43]. In support of our results, other reports showed that S100A8 and S100A9 were among the genes upregulated in SCC in comparison to normal skin [47]. Moreover, S100A8 and S100A9 were found to be upregulated in defined parts of dysplasia/cancer regions and in the invasion nests in SCC sections [48].

In summary, RAGE and its ligand S100A8/A9 seem active in SCC both in the setting of immunocompetence and immunosuppression, where immunosuppressive agents do not subdue the RAGE-S100A8/A9 axis but might potentially accentuate it.

To investigate the functional role of S100A8/A9 and RAGE, we generated primary keratinocyte cell cultures derived from normal skin and from lesions of patients with in situ (AK) or invasive SCC. Similar to other reports, we observed that low, rather than high concentrations of exogenous S100A8/A9 were sufficient to induce cellular proliferation [49, 50]. A potential function of S100A8/A9 as a driving factor for cell proliferation was reported in human breast cancer cells [19] and neonatal keratinocytes [51]. Correspondingly, we see S100A8/A9 as a driving factor for keratinocyte proliferation in human normal skin and SCC. Exogenous S100A8/A9 induced higher proliferation in normal keratinocytes in comparison to SCC keratinocytes unrelated to differential RAGE expression. We summarize that a differential dose-response relationship between the expression level of RAGE and S100A8/A9 and proliferation is hard to demonstrate due to several confounding factors such as origin of cells, number of cell passages, difference in cell signaling machinery between normal keratinocytes and different SCCs studied. Importantly, RAGE receptor blocking abolished the proliferative effect of exogenous S100A8/A9, indicating a critical role for RAGE. The possibility of additional receptors for S100A8/A9 on keratinocytes being involved such as TLR4 remains, as the secreted amount of S100A8/A9 from SCC cells was significantly higher than the decrease of the proliferation rate after RAGE blockade. However, we did not detect a decrease of cellular proliferation after the blockade of TLR4 by a specific blocking antibody. Our results correlate with other published data, where a relation between tumor growth and RAGE/S100A8/A9 axis was reported [15, 28]. Local production of S100A8/A9 within the epidermis may thus be a driving force for the proliferation of keratinocytes. Next to proliferation, our observation of increased migration suggests a role of S100A8/A9 in keratinocyte motility and a potential invasion.

Normal and SCC-derived keratinocytes showed distinct phosphorylation of JNK after S100A8/A9 stimulation, which is probably due to a different response to S100A8/A9, based on their origin (tumor vs. normal cells). Studies concerning the role of RAGE-S100A8/A9 axis in the induction of MAPK, JNK or p38 pathways show that depending on the cell type, a differential phosphorylation pattern of these kinases is observed. In breast and colon cancer cells, S100A8/A9 is found to induce phosphorylation of ERK and JNK [19, 35, 52]. These results consider a role for ERK phosphorylation in RAGE-S100A8/A9 axis. Similarly, we observed phosphorylation of ERK in both normal and SCC keratinocytes and suggest that it may contribute to the proliferation and migration after S100A8/A9 stimulation. It is also possible that this kinase profile is due to a crosstalk between the MAPK, JNK and p38 pathways, regulating the balance between apoptosis (p38, JNK) and proliferation (ERK1/2) [53].

S100A8/A9 may act as a growth factor similar to chemokines. A chemokine-like function of S100A8/A9 was described in terms of enhancement of leukocyte recruitment to inflammatory sites [54–56]. Keratinocytes may be the initial source for S100A8/A9, in turn attracting inflammatory cells. The effect of S100A8/A9 on cell growth may be mediated by cytokine induction. Recently, it was reported that exogenous S100A8/A9 induces the expression of inflammatory cytokines such as IL-8, IL-6 and TNF from neonatal keratinocytes [51] and macrophages [31, 34, 57]. While we hypothesize that S100A8/A9 influences the migration and invasion in SCC, its effect on metastasis remains controversial. Some reports show an inhibitory effect of S100A8/A9 on metastasis of human cervical cancer cells [58]. Clarifying the role of S100A8/A9 in the metastasis of SCC will be an important next step.

Organ transplant recipients suffer from SCC with a 60- to 100-fold increased incidence compared to the general population. In spite of their chronic immunosuppressive medication, a considerable amount of inflammation can still be observed in the local tumor microenvironment [4]. While low-level inflammation still occurs in the setting of chronic immunosuppression, tumor rejection by the immune system is impaired in organ transplant recipients, reflected by changes in the inflammatory microenvironment of SCC in OTR [59], [5]. We observed that the expression levels of RAGE and S100A8/A9 in OTR were still increased in comparison to healthy skin. It is tempting to speculate that S100A8/A9 induced by UV damage in sun-exposed skin could drive keratinocytes via RAGE to proliferate and migrate, eventually contributing to the greatly increased SCC formation in OTR.

In summary, we present evidence for a direct effect of S100A8/A9 on the proliferation and migration of normal human keratinocytes and keratinocytes originating directly from SCC lesions, suggesting a prominent role of S100A8/A9 on SCC formation, with RAGE as important target. This role of S100A8/A9 seems maintained in OTR. Targeting S100A8/A9 or RAGE may be of clinical benefit for SCC prevention and treatment.

## Materials and Methods

### Ethical considerations

The use of clinically indicated biopsy material for the study was approved by the ethical committee of the Canton of Zürich, Switzerland and was previously published: [60–64]

### Tissue samples

Invasive SCC from organ transplant recipients (n = 13) and immunocompetent patients (n = 19) as well as in-situ SCC from OTRs and immunocompetent patients (1 and 5 respectively) were obtained at the time of surgery. Normal skin was obtained from abdominoplastic reconstructive surgery (n = 5). All specimens’ diagnoses were confirmed by a board-certified dermatohistopathologist. 4mm punch biopsies from the SCC or normal skin area were placed in preheated PBS at 60°C for 45 seconds, and then chilled on ice in for 1 minute, followed by mechanical separation of epidermis and dermis. The epidermis was then homogenized in TRIzol Reagent (I*nvitrogen*, *Basel*, *Switzerland*), and stored at −80°C

#### RNA extraction and reverse transcription

Total RNA was isolated using TRIzol Reagent following the instructions provided by the manufacturer. cDNA was synthesized using a Reverse Transcription System kit (*Promega*, *Dübendorf*, *Switzerland*) following the protocol provided. After 1:4 dilution with sterile water, cDNA was stored at −20°C and used as template for subsequent quantitative real-time polymerase chain reaction (RT-PCR).

### Immunohistochemical analysis (IHC)

RAGE and S100A8/A9 protein expression was analyzed in formalin-fixed paraffin-embedded skin samples obtained from the archives of the Dermatology Department of University Hospital Zurich. The expression was tested in in-situ and invasive forms of SCC derived from OTRs and immunocompetent patients (5 samples per group)

Four μm thick sections were deparaffinized by 2x10 min incubation in Xylol and then rehydrated in a descending ethanol series (from 100% ethanol to PBS). For antigen retrieval, citrate buffer pH 6 (0.05% Tween 20) was pre-heated to 95°C, and the sections were kept at this temperature for 15 min. They were then allowed to slowly cool down to room temperature, and were then transferred to PBS. Blocking of nonspecific antibody binding was achieved by incubating the sections in PBS containing 2.5% of normal rabbit serum for RAGE staining or 2.5% normal goat serum for S100A8/A9. The staining was performed using anti-human RAGE polyclonal goat antibody (*R&D Systems Europe LtD*) and anti-human S100A8/A9 mouse monoclonal antibody (clone #27E10) (*BMA Biomedicals*, *Augst*, *Switzerland*) in a working dilution 1:50 (anti-RAGE) and 1:100 (anti-S100A8/9) in Antibody Diluent (*Dako*, *Hamburg*, *Germany*). Overnight incubation was performed in a humid chamber at 4°C. For detection of antibody binding to S100A8/A9 the Dako REAL Detection System AP/RED was used, and for detection of antibody biding to RAGE the Vectastain ABC-APKit (*Vector Laboratories*, *Burlingame*, *CA*, *USA*) was used according the manufacturers` protocol. The slides were briefly soaked in hematoxylin and then left under running tap water for 10 min. The slides were mounted with Mounting Medium (*Dako*).

### Generation of primary keratinocyte cultures derived from healthy individuals and SCC patients

Single keratinocytes were isolated from 3–4mm punch biopsies following the standard protocol for generation of primary keratinocyte cultures (*CELLnTEC*, *Bern*, *Switzerland*). For sufficient separation of the epidermis and dermis the punch biopsies were incubate overnight at 4°C in keratinocyte medium (Progenitor Cell Targeted (PCT) epidermal keratinocyte medium CnT07 (*CELLnTec*) with Dispase II (*Roche*). Afterwards the epidermal part of the skin was incubated in trypsin (room temperature, 30 min) to insure efficient isolation of single keratinocytes. Freshly isolated cells were washed with medium and then transferred into CnT07 medium for a continuous incubation (37°C, 5%CO2).

### RNA extraction and Real Time PCR

RNA was extracted using the TRIzol reagent (Invitrogen, Basel, Switzerland) following the protocol provided by the manufacturer. cDNA was synthesized from 0.5μg total RNA using a Reverse Transcription system kit (*Promega)*. Specific primers for RAGE (*Quantitect Primer assay*, *Quiagen AG*, *Hombrechtikon*, *Switzerland*), S100A8 (fwd GGGAATTTCCATGCCGTCT, rev CCTTTTTCCTGATATACTGAGGAC), S100A9 (fwd CTGTGTGGCTCCTCGGCT, rev GCGTTCCAGCTGCGACAT), 36B4 (fwd GCAATGTTGCCAGTGTCTGT, rev GCCTTGACCTTTTCAGCAAG) and K14 (*Quantitect Primer assay*, *Quiagen*) were used. Real Time PCR was performed using Light Cycler FastStart DNA Master Sybr Green 1 kit (*Roche*, *Switzerland*).

### Statistical analysis

Statistical analysis was performed using Microsoft Excel 2000 and GraphPad Prism 5.0 for Windows. The gene expression by real-time RT-PCR was quantified using the comparative threshold cycle (Ct) method [65]. Statistical evaluation was performed using the 2 way and one-way ANOVA test followed by Bonferroni’s Multiple Comparison Test, or t-test. P values less than 0.05 were considered statistically significant.

### Flow cytometry

#### FACS analysis

The assessment of the surface RAGE expression after induction with S100A8/A9 was performed using FACS analysis. Cells were detached using 4mM ETDA in PBS, washed with PBS, incubated with a specific goat anti-human RAGE antibody (*R&D Systems)* and visualized by specific donkey-anti goat FITC labeled secondary antibody (*Santa Cruz*, *Biotechnology*) in PBS at 4°C for 1h under gentle shaking conditions. The analysis was performed on 10 000 gated live cells together with a control (unstained and the secondary-FITC conjugated antibody) using FACSDIVA software (*BD Biosciences*, *Basel*, *Switzerland*).

Double staining for RAGE and BrdU was performed using the specific RAGE antibody and specific mouse anti BrdU antibody (*Millipore*). Before the staining cells were fixed with 4% PFA for 20min, followed by permeabilization with 0.5% Triton X, washed 3 times and proceeded for staining.

### BrdU proliferation assay

Cells were seeded in 96 well plates in serum-free CnT07 medium in a cell density of 4.10^3^/well. Incubation with purified S100A8/A9 protein was performed using concentrations between 0.01–1ug/ml for 24h. Blocking goat anti-human RAGE antibody (*R&D systems*) was added 1h prior to S100A8/A9 exposure. Cell proliferation was measured using the BrdU proliferation assay (*Millipore*) according to the manufacturer`s instruction. The percentage of cell proliferation was calculated using the equation: (mean OD of treated cells/mean OD of control cells) X 100. Purified S100A8/A9 protein was provided by Thomas Vogl, (Institute for Immunology, University of Münster, Germany), [31, 66]. Human S100A8/A9 was extracted from human granulocytes (c = 1,39 mg/ml, buffer: HBS, pH 7,4; source: granulocytes from 12 human buffy coats). No endotoxin contamination was detected by Limulus assay (LAL). The purity of the protein was confirmed by Coomassie blue staining after SDS electrophoresis.

### Immunoblotting

Normal and SCC-derived keratinocytes were grown in 6cm dishes in 60% confluence. Afterwards cells were stimulated with S100A8/A9 (10ng/ml) for different time intervals (0–60 min). Cells were lysed in RIPA buffer (*conventional recipe)*. Cell lysates were collected after each time interval and subjected to PAGE, followed by immunoblotting. The phosphorylation of ERK1/2, p38 and SAPK/JNK was detected by specific rabbit anti phospho-ERK1/2, -p38 and -SAPK JNK antibodies (*Cell Signaling Technology)*. The non-phosphorylated form of the kinases were detected using specific rabbit anti- ERK1/2, p38 (*Cell SignallingTechnology) and anti SAPK/JNK (Santa Cruz*, *Biotechnology)*. The loading control actin was detected by specific anti actin antibody (*Santa Cruz*, *Biotechnology)*.

RAGE expression in normal and SCC keratinocytes was confirmed by using of specific human anti RAGE antibody (*Millipore*).

### RNA knockdown study

Primary keratinocytes were infected with RAGE sh lentiviral particals (sc-36374-v) and control sh viral particals (sc-108080), following the protocol conditions (*Santa Cruz Biotechnology*). Positive clones were selected by puromycine selection. Stimulation of shRAGE and sh control expressing cells with S100A8/A9 protein was performed when the cells have reached an appropriate density. For determination of the effect of RAGE knockdown on cellular proliferation with a following S100A8/A9 stimulation, BrdU proliferation assay was performed.

### Scratch assay

Normal and SCC-derived keratinocytes were grown in 24 well plates until they reached full confluence and starved for further 15 hours in basal keratinocyte medium without supplements (*CELLnTEC*). Scratches were performed using a blue pipette tip. Cells were washed afterwards 2 times with basal keratinocyte medium. Exposure to exogenous S100A8/A9 was performed in a dose dependent manner using concentrations between 0.01–1μg/ml. Exposure was performed in triplicates for each concentration and control cells. After 15 and 24 hours, respectively, cells were fixed with 4% Paraformaldehyde (*Merck*, *Dietikon*, *Switzerland*), stained with Diff-Quik kit (*Medion-Diagnostics AG*, *Düdingen*, *Switzerland*), and counted in the whole scratch. The percentage of migrated cells was calculated as % of control.

## Supporting Information

S1 Fig RAGE expression in normal and SCC-derived keratinocytes. a: Surface and total expression of RAGE in normal keratinocytes. Surface RAGE expression was analyzed by FACS using specific anti RAGE antibody and FITC-conjugated secondary antibody. For intracellular staining cells were permeabilized (4%PFA) and blocked (1% BSA) prior staining with the specific RAGE antibody. As a control isotype control antibody was used. b: RAGE expression in normal and SCC derived keratinocytes. Cell lysates from primary normal and SCC keratinocytes were set to SDS PAGE electrophoresis and western blotting was performed using specific primary anti RAGE and secondary HRP-conjugated antibodies. As a loading control additional staining against actin was performed using specific anti actin antibody.
(TIF)

S2 Fig Densitometrical quantification of phosphorylated ERK, JNK and p38 proteins versus control. a: Spot density-based quantification of pERK1/2 versus ERK1/2 and versus loading control (actin). b: Spot density-based quantification of pSAPK/JNK (pp54/p46) versus SAPK/JNK and versus loading control (actin). c: Spot density-based quantification of pp38 versus p38 and versus loading control (actin).
(TIF)

S3 Fig RAGE expression in normal keratinocytes after RAGE knockdown by lentiviral shRNA. The RAGE expression after knockdown was analyzed on transcriptional level by qPCR using specific primers for RAGE and compared to sh control.
(TIF)

S4 Fig S100A8/A9 protein purification. S100A8/A9 has been extracted from granulocytes of buffy coats. The purity, quantity and quantity of the extracted S100A8/A9 were analyzed by Coomasie blue staining after SDS gel electrophoresis.
(TIF)
