# Supplementary figures and images for: S100A8/A9 Stimulates Keratinocyte Proliferation in the Development of Squamous Cell Carcinoma of the Skin via the Receptor for Advanced Glycation-End Products
Source: PLoS One. 2015 Mar 26;10(3):e0120971. doi: 10.1371/journal.pone.0120971 (PMC4374726; doi:10.1371/journal.pone.0120971)

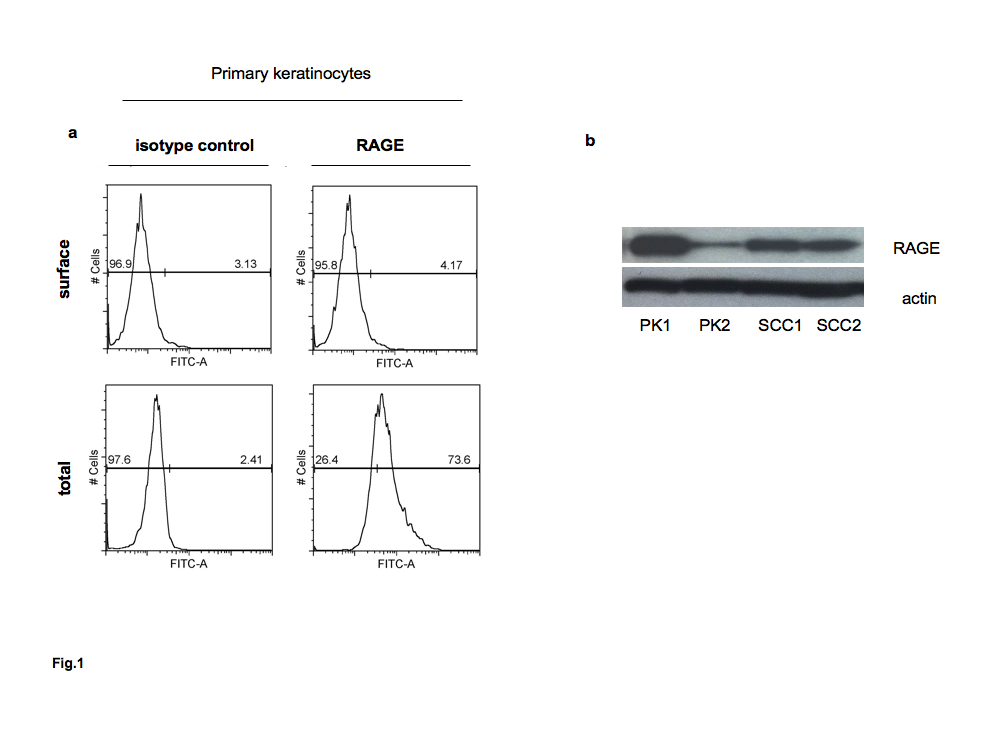

Supplement: S1 Fig — a: Surface and total expression of RAGE in normal keratinocytes. Surface RAGE expression was analyzed by FACS using specific anti RAGE antibody and FITC-conjugated secondary antibody. For intracellular staining cells were permeabilized (4%PFA) and blocked (1% BSA) prior staining with the specific RAGE antibody. As a control isotype control antibody was used. b: RAGE expression in normal and SCC derived keratinocytes. Cell lysates from primary normal and SCC keratinocytes were set to SDS PAGE electrophoresis and western blotting was performed using specific primary anti RAGE and secondary HRP-conjugated antibodies. As a loading control additional staining against actin was performed using specific anti actin antibody. (TIF) [file pone.0120971.s001.tif]

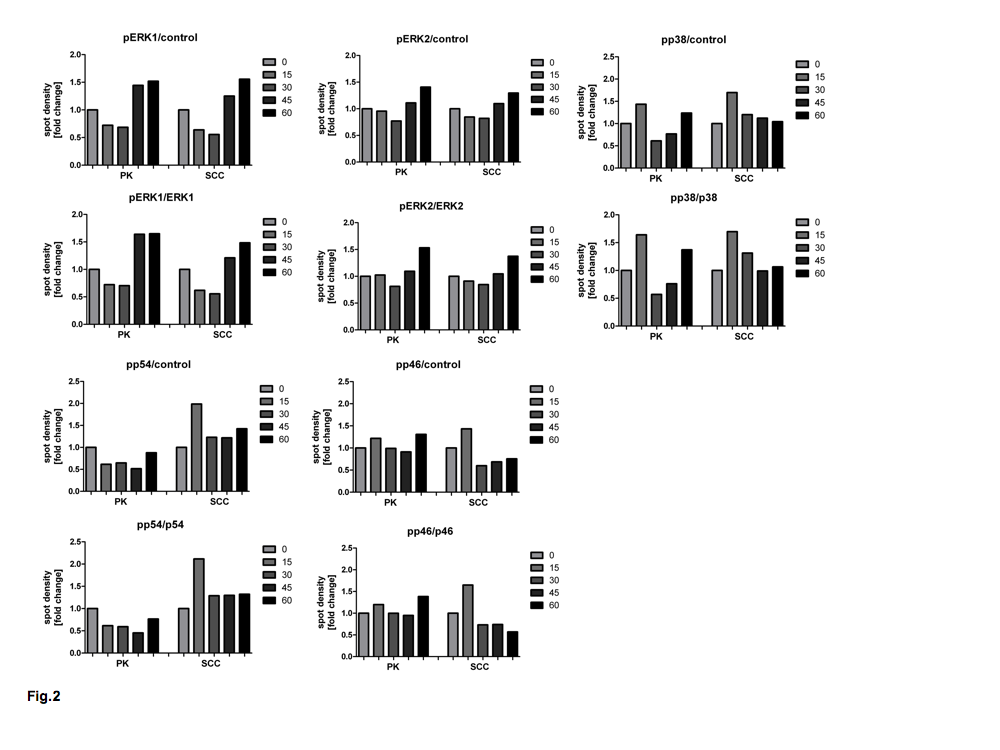

Supplement: S2 Fig — a: Spot density-based quantification of pERK1/2 versus ERK1/2 and versus loading control (actin). b: Spot density-based quantification of pSAPK/JNK (pp54/p46) versus SAPK/JNK and versus loading control (actin). c: Spot density-based quantification of pp38 versus p38 and versus loading control (actin). (TIF) [file pone.0120971.s002.tif]

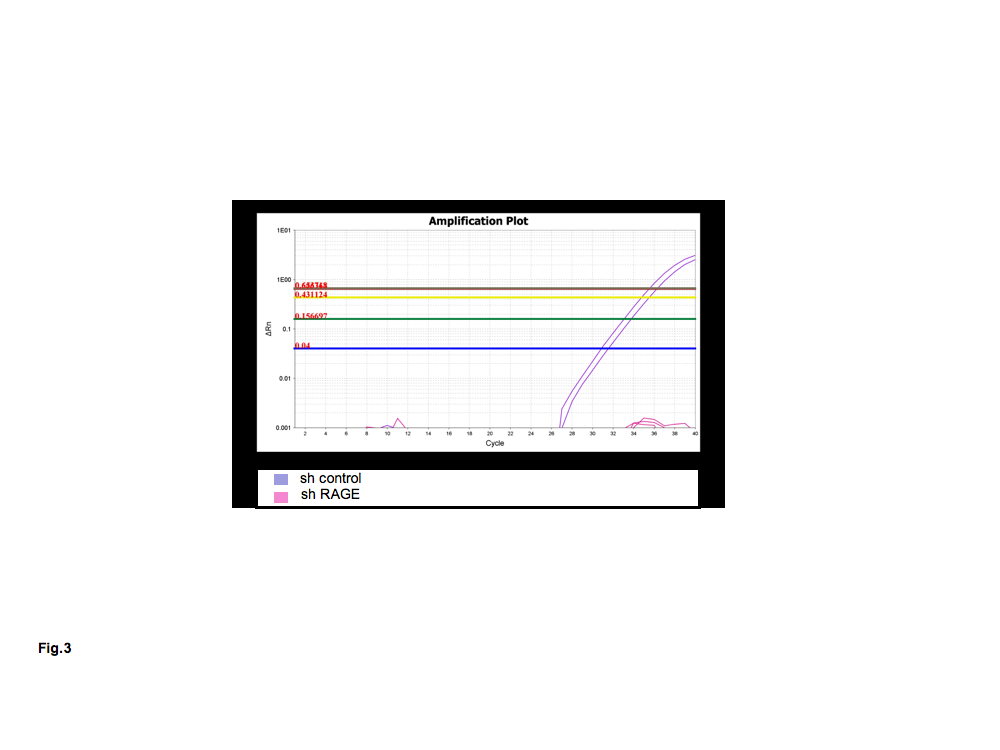

Supplement: S3 Fig — The RAGE expression after knockdown was analyzed on transcriptional level by qPCR using specific primers for RAGE and compared to sh control. (TIF) [file pone.0120971.s003.tif]

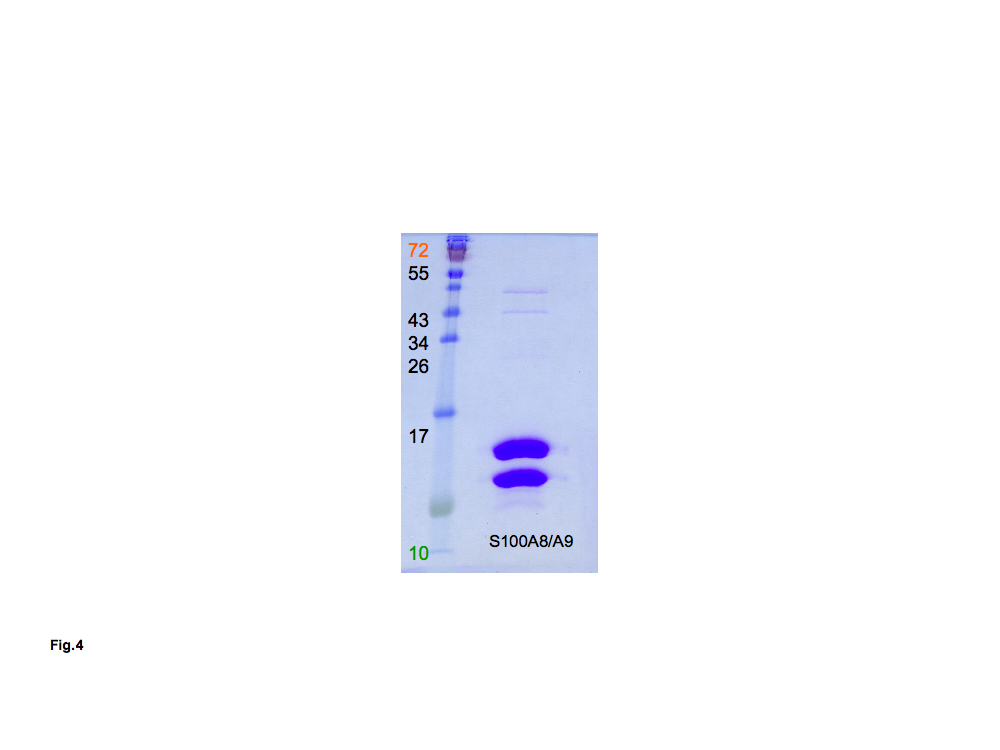

Supplement: S4 Fig — S100A8/A9 has been extracted from granulocytes of buffy coats. The purity, quantity and quantity of the extracted S100A8/A9 were analyzed by Coomasie blue staining after SDS gel electrophoresis. (TIF) [file pone.0120971.s004.tif]
